# Automatic Stimulus-Induced Medial Premotor Cortex Activation without Perception or Action

**DOI:** 10.1371/journal.pone.0016613

**Published:** 2011-02-02

**Authors:** Kevin D'Ostilio, Gaëtan Garraux

**Affiliations:** Cyclotron Research Center, University of Liege, Liege, Belgium; French National Centre for Scientific Research, France

## Abstract

Who has ever been surprised to return to the bowl of salted peanuts without realizing it, even after having eating a moderate number and deciding to stop? Using rapid event-related functional magnetic resonance imaging (fMRI) in healthy volunteers, we investigated the neural correlates of automatic processes induced by subliminal stimuli. We demonstrated that the automatic activation of motor programs elicited unconsciously in the medial premotor cortex was normally restricted to specific contexts set by the environment, but can occur below the threshold of awareness even when no movement was executed. This novel finding expands our view on brain mechanisms underlying unconscious motor control and provides new evidence that activation of the motor preparation system and consciousness are not obligatory linked.

## Introduction

We have substantial evidence suggesting that what man perceives and reacts to is not necessarily available to consciousness [1], [2], [3]. Everybody has already experienced situations in which an unintentional behavior is nevertheless executed. Who has ever been surprised to return to the bowl of salted peanuts without realizing it, even after having eaten a moderate number and deciding to stop? The build-up of a readiness potential recorded from the scalp hundreds of milliseconds before the conscious intention to initiate a movement [4] has been taken as evidence for a role of the medial premotor cortex (mPMC) [5], [6] in unconscious motor programming. This has also been supported by a perturbation of free will following a temporary [7] or permanent [8] lesion of this region. In another series of brain mapping experiments, modifications in reaction time induced by subliminal stimuli (i.e., that are presented below the threshold of awareness) have been associated with activity changes up to the level of the primary motor cortices, suggesting again that an activation of motor programs can be elicited unconsciously [9], [10], [11]. However, what happens in the brain when incoming flow of information endlessly generated by our environment is not systematically followed by a motor response.

Here, we hypothesized that this pattern of brain response can occur even when no movement is executed. To address the question of unconscious and automatic motor activation, we conducted a rapid event-related fMRI experiment during which participants performed a subliminal visuo-motor priming task.

## Materials and Methods

### Ethics statement

All procedures were executed in compliance with relevant laws and institutional guidelines and were approved by the ethics committee of the Faculty of Medicine, University of Liège, Belgium. Subjects gave written informed consent before participation.

### Participants

Twenty-four young healthy adults (6 men) participated in the fMRI experiment. Age ranged from 19 to 27 years, with a mean±SD of 22±2 years. All were right handed, had normal or corrected-to-normal vision, and were naive to the purpose of the experiment.

### Procedure

**Figure 1:**
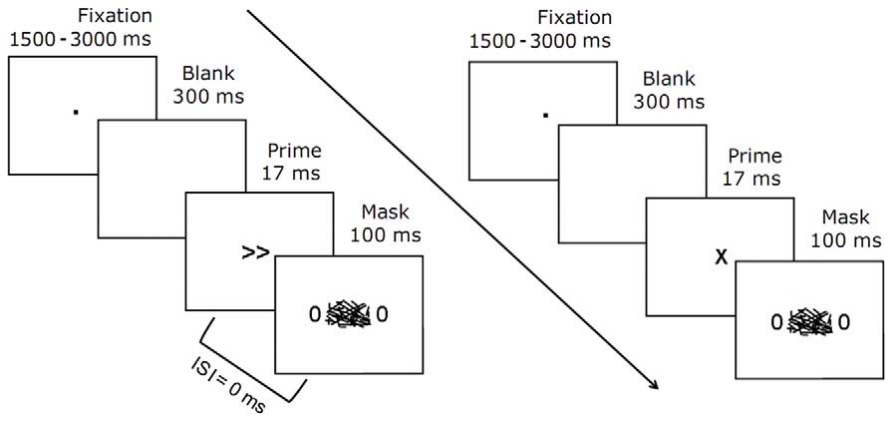
Example of no-response trials of the masked prime task. Double “0” signs appeared together with the mask directly after the prime presentation (interstimulus interval of 0 ms). The only difference in the two conditions was the 17-ms-unperceived-prime.

Prior to fMRI, participants were trained to make rapid button presses with the left or right hand in response to the display of double leftward (≪≪) or rightward (≫≫) arrows. In the scanner, we measured reaction time (RT) needed to respond to target arrows in three experimental conditions (compatible, incompatible and neutral) that differed by the context set by the physical properties of a subliminal stimulus presented immediately before the target stimulus. Unbeknownst to the subjects, the subliminal stimulus was either an “X” sign or a double arrow pointing to the same or opposite direction as compared with the target stimulus. Additionally, the target stimuli, which appeared on each side of the mask, were either two double arrows (response trials) or two “0” signs (no-response trials) (**Fig. 1**). Each trial started with a central fixation dot. Its display was pseudo-randomly jittered between 1500 and 3000 milliseconds. The fixation dot was immediately followed by a blank screen and a 17 ms-duration prime-stimulus, immediately replaced by a backward mask. The mask appeared for 100 ms and consisted of 30 randomly oriented lines that changed for each trial. Thus, the experiment included 40 compatible (same direction for prime and target arrows), 40 incompatible (opposite direction), 40 neutral (non-arrow prime stimuli followed by target arrows), 40 primed no-response (arrow prime stimuli but no target arrow) and 40 neutral no-response trials (non-arrow prime stimuli and no target arrow) and also 40 null events (fixation cross). The RT measures serves to interpret fMRI results of no-response trials.

The level of prime perception was assessed by means of two different identification tasks, administered in the scanner. The first one, including 23 supplementary subjects, consisted of a staircase procedure with only left and right pointing arrows. The target stimulus was a question mark displayed from 1 to 1.7 seconds after the mask. The task always started with a 167 ms display trial and prime display was shortened by steps of 17 ms whenever participants gave a correct response and prolonged by 34 ms after an incorrect response. In the second identification task, using the same display as the main experiment, 10 new subjects were asked to judge whether the 17-ms-prime was a left/right arrow (80 trials) or a neutral “X” sign (40 trials).

### Behavioral data analysis

We performed t-tests to compare the accuracy rate with chance performance in the identification task. For the main task, we performed a repeated measures ANOVA on mean reaction time (RT) with the condition as an intra-subject factor.

### fMRI

BOLD fMRI data were acquired on a 3 Tesla scanner (Siemens, Allegra, Erlangen, Germany) using a T2* sensitive gradient echo EPI sequence (TR = 1170 ms, TE = 30 ms, FA = 90°, matrix size 64×64×20, voxel size = 3.4×3.4×6 mm3). Twenty 3-mm-thick slices were acquired, with a distance factor of 30%, covering nearly the whole brain. For each session, the first eight volumes, acquired before stimulus presentation, were discarded to allow for T1 saturation effects. Head movement was minimized by restraining the subject's head using a vacuum cushion. Stimuli were displayed on a screen positioned at the rear of the scanner, which the subject could comfortably see through a mirror mounted on the standard head coil. A high resolution structural images was obtained using a T1-weighted 3D MDEFT sequence (TR  = 7.92 ms, TE  = 2.4 ms, FA = 15°, matrix size  = 224×256×176, voxel size = 1×1×1 mm3).

Data were preprocessed and analyzed using SPM8 (Wellcome Department of Imaging Neuroscience, http://www.fil.ion.ucl.ac.uk/spm) implemented in MATLAB 7.4.0 (Mathworks Inc., Sherbom, MA). Images of each individual subject were first corrected for slice timing and realigned (motion corrected). Imaging data from one participant was excluded from data analysis because of significant head movement artifacts. The mean EPI image was spatially coregistered to the anatomical MRI image and coregistration parameters were applied to the realigned BOLD time series. Individual anatomical MRIs were spatially normalized into MNI space (Montreal Neurological Institute, http://www.bic.mni.mcgill.ca) and the normalization parameters were subsequently applied to the individually coregistered BOLD times series, which was then resliced to a voxel size of 2×2×2 mm, and smoothed using an 6 mm FWHM Gaussian kernel. Each event was convolved with a canonical hemodynamic response function and its time and dispersion derivatives. We used the generalized linear model to model the intensity level of each voxel as a linear combination, for each subject and event. We constructed for each individual subject statistical contrasts by subtracting null events from each condition. In this fMRI analysis, we focused on no-response trials, the others being used for another study. In fact, the other conditions are not useful to study this unconscious process because of the overlap between prime-induced automatic motor activation and response to target that might involve the same regions. Individual contrasts were then entered in a second-level-analysis (random-effect analysis). We also performed a conjunction analysis in order to assess the overlap between overt and covert premotor activity. We used a cluster significance threshold of *P*<0.05 corrected for multiple comparisons across the brain volume.

## Results and Discussion

In the first identification task, performance was at chance level for primes presented for 17 ms (mean percentage correct responses  = 54,7%, *t*(46)  = 1.46, p>0.1). In the second identification task, performance was also at chance level (mean percentage correct responses  = 31,7%, *t*(9)  = 0.79, p>0.4). These results support the view that prime stimuli were unlikely to be consciously perceived during no-response trials.

As expected, we replicated the classical positive compatibility effect (PCE), namely shorter reaction times in compatible trials (mean RT: 369±38 ms) than in incompatible trials (mean RT: 383±30 ms) in comparison to neutral trials (mean RT: 375±38 ms) (main effect: *F*(2,46)  = 7.72; p<0.01). The gain in motor performance in the compatible condition results from an automatic, context-specific, selection of the correct motor program [12], [13].

**Figure 2:**
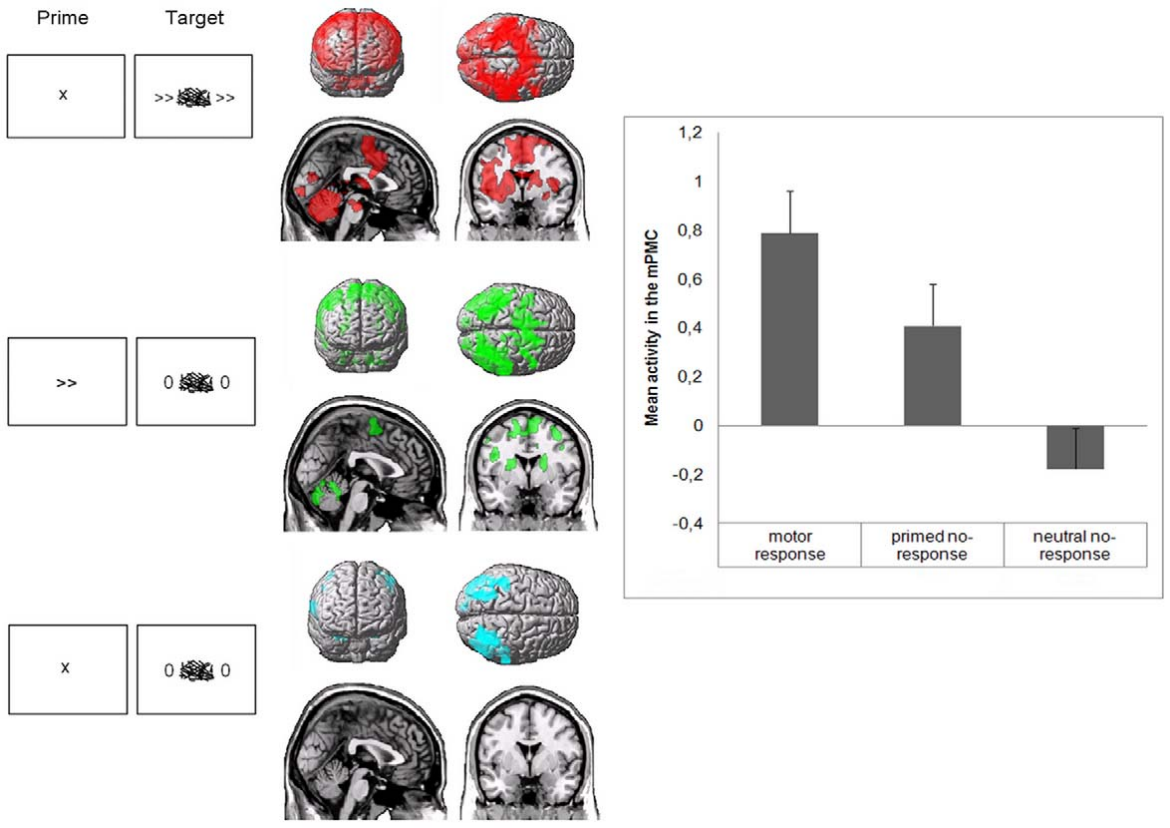
fMRI results. *Left:* Activation in the motor response condition (red), the primed no-response condition (green) and the neutral no-response condition (blue) minus baseline. In the neutral no-response condition, activation was mainly restricted to posterior brain areas. In the primed no-response condition, this activation extended to rostral brain regions classically involved in motor preparation. *Right:* Comparison of mPMC activity in motor response and no-response conditions. Error bars represents the standard error. Paired t-test shows a greater activity in the mPMC for the primed no-response condition in comparison to the neutral no-response condition (*P* = 0.046 corrected).

During fMRI, responded trials were randomly intermixed with non-responded trials, where target stimuli were replaced by a double “0” sign, under the assumption that the above effects were similar in both trial types. Imaging analysis of these non-responded trials revealed brain activation mainly restricted to posterior brain areas involved in visual perception, when the context was set by neutral prime stimuli irrelevant to the intended motor response. This activation spread to the mPMC and other areas involved in motor programming such as the striatum (voxel *P* = 0.01, cluster *P*<0.05 corrected for whole-brain multiple comparisons; **Fig. 2**) when the context was represented by subliminal arrow stimuli. This differential activity was significant in the mPMC (paired t-test, *P* = 0.046 corrected for whole-brain multiple comparisons). This context-specific medial premotor activation showed a significant overlap with that observed during responded trials as demonstrated by a conjunction analysis between the two conditions. In fact, the mPMC was activated in both the motor response and the primed no-response condition (cluster level *P* corrected  = 0.00003) but not in the neutral no-response condition (cluster level *P* uncorr. >0.25).

For the first time, our result uncovers an important mechanism by which stimuli endlessly generated by our environment can potentially influence our behavior even if they are not consciously perceived. This novel finding strongly supports a central role for the mPMC in unconscious motor actions. Compared with previous work, the crucial points here are that the motor preparation system activation is specific for the context set by the environment (i.e., physical properties of the prime stimulus) and can be elicited even when no motor response is executed. The present result also provides an undisputable demonstration that mPMC activity and consciousness are not necessarily linked. This expands our current understanding of brain mechanisms underlying unconscious motor control and suggests care in the interpretation of neuroimaging studies in patients with no sign of awareness at standard clinical exam. The activation of the mPMC in those patients when asked to imagine themselves playing tennis has been interpreted either as a preserved intention to collaborate [14] or an automatic mechanism triggered by the mere exposure to specific stimuli [15]. The latter has been demonstrated here as being entirely possible.
